# Assessment of ‘Cool’ and ‘Hot’ Executive Skills in Children with ADHD: The Role of Performance Measures and Behavioral Ratings

**DOI:** 10.3390/ejihpe12110116

**Published:** 2022-11-17

**Authors:** Andreia S. Veloso, Selene G. Vicente, Marisa G. Filipe

**Affiliations:** 1 Centre for Psychology at University of Porto, Faculty of Psychology and Education Sciences, Rua Alfredo Allen, 4200-135 Porto, Portugal; 2 Center for Linguistics, School of Arts and Humanities, University of Lisbon, Alameda da Universidade, 1600-214 Lisboa, Portugal

**Keywords:** attention deficit/hyperactivity disorder, executive functions, hot executive functions, cool executive functions, children, performance-based measures, behavioral ratings

## Abstract

Executive dysfunction is an underlying characteristic of Attention Deficit/Hyperactivity Disorder (ADHD). Therefore, this study explored which measures of executive functions (EF) may lead to a better diagnostic prediction and evaluated whether participants were adequately assigned to the ADHD group based on the identified predictors. Seventeen 6- to 10-year-old children with ADHD were matched with 17 typically developing peers (TD) by age, gender, and non-verbal intelligence. Performance-based measures and behavior ratings of ‘cool’ and ‘hot’ EF were used. As expected, there was a significant group effect on the linear combination of measures, indicating that children with ADHD showed significant difficulties with EF compared to the TD group. In fact, significant differences were found in measures of short-term and working memory, planning, delay aversion, and EF-related behaviors, as reported by parents and teachers. However, the discriminant function analysis only revealed three significant predictors: the General Executive Composite of the Behavior Rating Inventory of Executive Function (Parent and Teacher Forms) and the Delay of Gratification Task, with 97.1% correct classifications. These findings highlight the importance and contribution of both behavioral ratings and ‘hot’ measures of EF for the characterization of ADHD in children.

## 1. Introduction

Attention Deficit/Hyperactivity Disorder (ADHD) is a childhood-onset neurodevelopmental disorder characterized by inappropriate levels of inattention, hyperactivity, and/or impulsivity [1]. With a worldwide prevalence of 5% [1], this disorder affects the child’s daily functioning in areas such as academic performance and social/interpersonal relationships [2,3]. ADHD is typically diagnosed during school years and approximately 80% of diagnosed children show persistent symptoms throughout adolescence and adulthood [4,5].

Distinct features are implicated in the etiology of ADHD, particularly genetic and environmental factors (prenatal, perinatal, and postnatal factors) [6]. The interaction of these factors creates a spectrum of neurobiological liability [6], prompting alterations within several neural networks and their underlying neuropsychological functions [7]. Consequently, ADHD is a highly comorbid disorder, with children frequently exhibiting cooccurrence of symptoms with Autism Spectrum Disorders, Learning Disabilities, Opposition Defiant Disorder, language problems, anxiety, depression, and sleep pathologies [8,9,10,11,12,13].

Numerous studies have been carried out to characterize ADHD from a neuropsychological point of view, and within these studies executive functions (EF) have been widely investigated [14,15,16,17,18,19,20,21,22,23,24,25,26,27]. Despite the vast literature on EF, there is no agreed-upon definition that fully captures the complexity of these processes [28]. Still, EF can be viewed as a multidimensional construct that refers to higher-order cognitive processes responsible for controlling and regulating several cognitive, emotional, and behavioral functions [29] (for a comprehensive review of EF definitions, see [28,30]). Encompassed within the EF construct are the concepts of attentional control, planning, cognitive flexibility (or set shifting), monitoring, goal setting, inhibition, problem solving, initiation of activity, and self-regulation [31,32,33,34]. These functions develop throughout childhood and adolescence and are paramount for daily functioning at the cognitive, behavioral, emotional, and social levels [31].

As several authors posit that executive dysfunction is an underlying characteristic of ADHD (e.g., [35,36]), EF have been extensively investigated in children with this diagnosis, and its assessment is usually carried out using measures of performance and/or behavioral ratings. While performance-based measures are characterized by standardized procedures administered by a trained researcher or clinician, in a structured environment, behavioral ratings assess the child’s behaviors associated with EF in everyday life from the point of view of an informant, usually a parent and/or teacher [37,38].

Although the evaluation of EF in children with ADHD using performance-based measures has proven to be heterogeneous, children with this diagnosis appear to have greater difficulties performing EF tasks compared to their typically developing peers. For example, a meta-analysis showed that children with ADHD demonstrate significant deficits in inhibitory control, vigilance, working memory, and planning (weighted mean effect size was 0.54; 95% CI = 0.51–0.57) [18]. Similarly, deficits in processing speed (e.g., [20,26]), sustained attention (e.g., [16,27]), and cognitive flexibility (e.g., [19,21]) have been reported. However, as discussed by Nigg et al. [39], it is possible that only a subgroup of children with ADHD present EF deficits. In fact, the authors found that 21% of children with ADHD do not have weaknesses in any outcome related to EF and merely 10% present difficulties in five or more EF tasks. According to the authors, only 35% to 50% of children with ADHD present a dysfunctional level of EF performance [39]. 

Furthermore, research has found broad EF difficulties when these functions are assessed through behavioral ratings. For example, Tan et al. [40] showed that participants with ADHD had significantly higher difficulties with inhibition, initiative, working memory, and planning/organization than their typically developing peers in one of the most widely used behavioral ratings of EF (i.e., Behavior Rating Inventory of Executive Function; BRIEF; [41]). Furthermore, according to the same study, children with ADHD also had significantly elevated ratings on all BRIEF main indices, pinpointing difficulties with EF-related behaviors and tasks in daily life [40]. Soriano-Ferrer et al. [42] obtained similar results as higher levels of difficulties across the BRIEF subscales were highlighted by parents and teachers. Other studies have reported comparable results (e.g., [17,43,44]). Thus, behavioral EF measures appear to have a higher sensitivity to deficits in executive functioning compared to performance-based measures [45].

The lack of convergence found between results obtained through performance-based and behavioral measures of EF led some authors to hypothesize that these two classes of instruments are assessing different levels of the same construct [38,45,46,47]. According to Toplak et al. [38], performance-based measures assess the optimal performance of the child in an extremely structured environment in which the examiner provides precise instructions and directions. As such, the results obtained through these measures may not be illustrative of the child’s daily functioning, possibly leading to inadequate detection of EF deficits [37]. Behavioral measures, on the other hand, evaluate the typical performance of the child by assessing its ability to pursue goals in nonstructured situations [38], possibly providing a more accurate depiction of the child’s EF abilities. Moreover, low correlations between these measures are often found and can further justify the distinctive results often reported in the literature (e.g., [29,47,48]). For example, McAuley et al. [47] found no significant associations between the BRIEF and performance-based measures of inhibition, monitoring, and working memory (Stop-Signal and N-Back tasks) in a sample of clinic-referred youth. Similarly, Mahone et al. [48] found no significant correlations between the BRIEF and measures of verbal fluency, planning, and attention in children with ADHD. Equivalent results have been found in other studies (e.g., [29]). However, the few studies that found significant associations between both types of measures (e.g., [17,49]) highlight the need for further research.

Although executive dysfunction appears to be present in children with ADHD, it does not seem sufficient to fully characterize the disorder from a neuropsychological perspective [50,51]. As such, the dual pathway model developed by Sonuga-Barke (2002) [52] emphasizes, along with executive dysfunction, the importance of motivation in understanding the disorder. Other authors refer to this as cognitive (‘cool’ EF) and affective/motivational processes (‘hot’ EF). According to this framework, ‘cool’ EF are characterized entirely by cognitive functions necessary for problem solving, while ‘hot’ EF relate to affective and motivational aspects of executive functioning necessary when a situation is emotionally significant [53]. Despite this distinction, these processes are interrelated [53,54,55]. Successful problem solving (‘cool’ EF) depends highly on one’s motivational and emotional influences [‘hot’ EF] and, to solve motivationally charged problems, it is necessary to reflect, contextualize, and analyse them abstractly [‘cool’ EF] [54,56]. Therefore, and as stated by Sonuga-Barke [52], the motivational style of the child, characterized by a higher sensitivity to rewards (i.e., delay aversion) that leads to actively evading delaying gratification [51,57], could further elucidate on the neuropsychological profile of individuals with ADHD. Moreover, these motivational aspects can also justify the differences found in the results obtained using performance and behavioral measures. According to Barkley [28], performance-based measures do not consider the motivational, social, and cultural aspects of EF present in daily life (i.e., ‘hot’ EF).

In line with this approach, studies have been showing that, when compared to typically developing peers, children with ADHD tend to prefer smaller immediate rewards over larger delayed ones [58]. For example, children with ADHD demonstrated a preference for immediate rewards of lower value by choosing larger rewards less frequently on the Choice-Delay Task [58]. Other studies and meta-analytic reviews found similar results (e.g., [57,59,60,61]). Therefore, despite progress in understanding the value of ‘hot’ EF for the characterization of ADHD, the need for approaches to unravel their importance is undeniable as they could have significant implications in intervention planning.

### Present Study

As it becomes essential to understand which EF measures are more advantageous to inform assessment practices, this study sought to characterize the EF profile of children with ADHD by comparing ‘cool’ (short-term and working memory, cognitive flexibility, verbal fluency, and planning) and ‘hot’ (delay aversion) aspects of EF through two types of measures: performance-based neuropsychological measures and behavioral ratings. Specifically, we intended to further the literature by assessing which measures are better predictors of group membership and evaluating the degree to which participants are correctly assigned to the group based on the identified predictors. Thus, our aim is to answer the following research questions:

(1) In which dimensions of EF do children with ADHD differ from children with typical development? (i.e., discriminant procedure);

(2) How can EF measures be combined to reliably separate groups? (i.e., classification procedure);

(3) Based on the classification procedure, what proportion of children was correctly identified and classified in their group? (i.e., the suitability of the classification procedure).

## 2. Materials and Methods

### 2.1. Participants

Thirty-four children (14 girls, 20 boys) from the North of Portugal—recruited either within school or clinical contexts—between the ages of 6 and 10 years participated in this study. All participants included in the clinical group had a confirmed diagnosis of ADHD-combined presentation, performed by an experienced child psychiatrist or pediatrician (clinical judgement based on information gathered from multiple informants and DSM 5 diagnostic criteria), and met diagnostic criteria on the Conners Rating Scale Revised—Short Form [62,63] according to parents. Children with ADHD were matched to a typically developing group (TD) by age, gender, and non-verbal intelligence (ADHD: *M* = 24.44; SD = 4.08; TD: *M* = 24.67; SD = 4.14; assessed with Ravens Colored Progressive Matrices, RCPM; [64,65]). Descriptive characteristics are summarized in Table 1. The eligibility criteria for both groups required the following: (a) absence of intellectual disability, (b) absence of a history of congenital or acquired neurologic disorders, traumatic brain injury, or epilepsy, (c) no report of sensory problems (e.g., vision and/or hearing), and (d) no comorbid diagnosis that may have confounded the study objectives (e.g., Opposition Defiant Disorder, Learning Disability, Autism Spectrum Disorder, Intellectual Disability).

### 2.2. Measures

The performance-based measures and behavioral rating described below were used to assess a set of abilities. Table 2 describes the specific domains evaluated by each measure.

#### 2.2.1. Ravens Colored Progressive Matrices (RCPM) [64,65]

This instrument was used to assess the child’s ability to analyze information and solve problems using visual reasoning, providing a score for nonverbal intelligence. This task consists of 36 problems divided into three series. Within each problem, the child is asked to select, out of six available options, the missing piece that best completes a patterned image. The outcome was the number of correctly solved problems.

#### 2.2.2. Digit Span Subtest—Wechsler Intelligence Scale for Children-III (WISC-III) [66,67]

The digits subtest was used to assess short-term and working memory. In this task, the child is instructed to repeat increasingly longer sequences of digits in forward and backward order. The outcome was the number of trials that were executed correctly.

#### 2.2.3. Trails—Coimbra Neuropsychological Assessment Battery (Bateria de Avaliação Neuropsicológica de Coimbra; BANC) [68]

Trails (Parts A and B) were used to assess selective, sustained attention, processing speed, and cognitive flexibility. Both parts of the Trails consist of numbers distributed on a sheet of paper. The participant must connect the numbers as fast as possible, without lifting the pencil from the paper. In Part A, the child is asked to connect the numbers in ascending order (from 1 to 25). In Part B, the child is asked to link the numbers in an ascending pattern with the added task of alternating between numbers and letters (e.g., 1-A-2-B-3-C). For these tasks, the result was the time (in seconds) that the child took to complete each ‘trail’.

#### 2.2.4. Tower—Coimbra Neuropsychological Assessment Battery (Bateria de Avaliação Neuropsicológica de Coimbra; BANC) [68]

The tower test was used to assess planning and problem solving. In this task, the child should transfer three colored balls (red, green, and blue) between three vertical pegs of progressively shorter height, from a prescribed start position to a target arrangement in a pre-established number of moves. The outcome was the number of problems correctly solved in the first trial.

#### 2.2.5. Delay of Gratification Task (DGT) [69]

Considering the duality of EF (i.e., ‘cool’ vs. ‘hot’ aspects), this task was intended to assess affective/motivational decision making. Within nine trials, children were able to choose between an immediate reward of lower value (i.e., “impulsive” choice) and a delayed reward of higher value (i.e., “rational” choice). For this study, pens, stickers, and candies were used as rewards. The outcome was the number of times the child chose the delayed reward.

#### 2.2.6. Behavior Rating Inventory of Executive Functions (BRIEF) [41]

The BRIEF is a questionnaire for parents and teachers to assess EF-associated behaviors in home and school settings. Each form is comprised of 86 items subdivided into eight scales that represent key aspects of EF: inhibit, shift, emotional control, initiate, working memory, plan/organize, organization of materials, and monitor. These scores allow the computation of two broader indexes (i.e., Behavioral Regulation Index and Metacognition) and an overall score (i.e., Global Executive Composite). In this study, a translation to Portuguese by Barbosa, Teles, and Vicente (2011) [61] was used. The outcome was the scale reflecting overall executive functioning (i.e., Global Executive Composite scores) from both parent and teacher reports.

### 2.3. Procedure

The study was approved by the Ethics Committee of the Faculty of Psychology and Education Sciences of the University of Porto, following the Declaration of Helsinki. Oral permission from the children and written informed consent from their parents were obtained. Subsequently, a semi-structured interview was conducted with parents to gather information about family functioning, family psychiatric history, child development, and medical history. Parents and teachers completed the rating scales at home, and children were individually assessed on performance-based measures, typically at two different moments, each with a duration of one hour. The order of application of the performance-based measures was as follows: Ravens Colored Progressive Matrices, Digit Span, Trails, Tower, and Delay of Gratification Task.

All participants with ADHD were asked to refrain from any pharmacological treatment 24 h before scheduled assessment sessions.

### 2.4. Statistical Approach

Both groups were equally sized and were matched by age, gender, and non-verbal intelligence. There were no missing data for any of the participants. Differences between groups in performance and behavioral measures were evaluated using Multivariate Analysis of Variance (MANOVA). Effect sizes were calculated using a partial eta square (*ηρ*^2^) [70]. The predictive value of the EF measures, both performance and behavioral, was assessed using a direct discriminant function analysis (DFA) to predict group membership.

#### Outliers and Normality

Some participants displayed extreme performance on individual scores in two specific tasks (Tower and Trails), therefore being classified as univariate outliers. As these were considered cases sampled from the intended population [63], each extreme and deviant score was changed to the next highest or lowest score that was not considered an outlier [64]. No multivariate outliers were found, resorting to the Mahalanobis distance, either before or after the outlier transformation was performed. Given the small sample size included in this study, a conservative alpha level of 0.001 was used to assess normality in the Shapiro–Wilk test [63].

## 3. Results

Table 1 displays the descriptive statistics for all variables between groups. There were no significant differences between groups in age or nonverbal intelligence.

### 3.1. Differences between Groups

A MANOVA was performed with group as a between-subject factor and the five outcomes derived from the performance-based measures (i.e., Digit Span, Trails: Part A, Trails: Part B, Tower, and Delay of Gratification Task) and the General Executive Composite (Parent and Teacher Form) as the dependent variables to investigate group differences in executive functioning. Preliminary assumption testing was conducted. There was a significant group effect on the combined dependent variables, *F*(7, 26) = 17.96, *p* < 0.001; Wilks Lambda = 0.17; *ηρ*^2^ = 0.83, indicating that children with ADHD showed lower performance and higher levels of executive behavioral dysfunction in the linear combination of EF measures compared to the TD group.

### 3.2. Prediction of Group Membership

**Figure 1:**
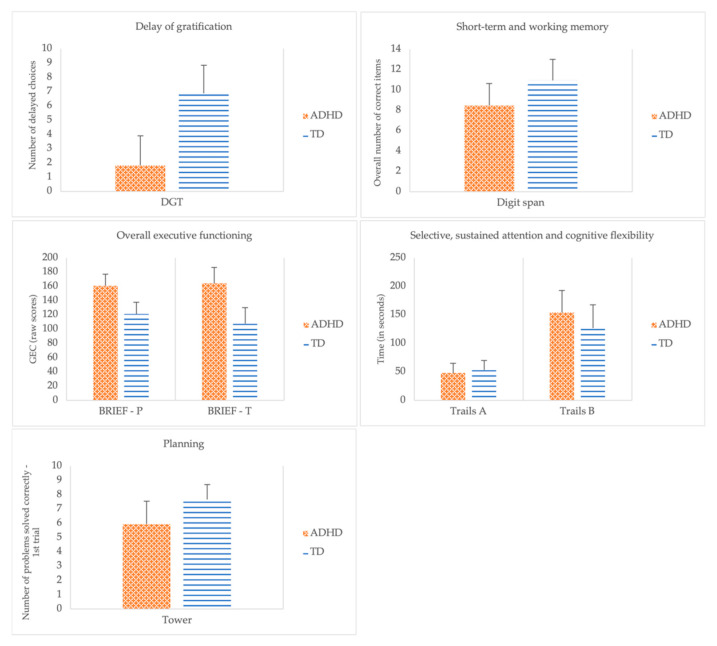
Group comparisons across performance and behavioral measures of executive functions.

A direct discriminant analysis was performed using the seven EF measures as predictors of group membership. Significant mean differences were observed for all the predictors (Digit Span: *F*(1, 32) = 11.76, *p* = 0.002; Wilks Lambda = 0.73; Tower: *F*(1, 32) = 13.46, *p* < 0.001; Wilks Lambda = 0.70; Delay of Gratification Task: *F*(1, 32) = 53.45, *p* < 0.001; Wilks Lambda = 0.37; BRIEF General Executive Composite—Parent Form: *F*(1, 32) = 40.46, *p* < 0.001; Wilks Lambda = 0.44; BRIEF General Executive Composite—Teacher Form: F(1, 32) = 71.81, *p* < 0.001; Wilks Lambda = 0.31) with the exception of Trails: Part A, *F*(1, 32) = 0.78, *p* = 0.383; Wilks Lambda = 0.98 and Trails: Part B, *F*(1, 32) = 3.91, *p* = 0.057; Wilks Lambda = 0.89. Figure 1 depicts the comparison between groups. The discriminant function revealed a significant association between groups and all predictors, accounting for 82.81% of the variability between groups. Although differences between groups were found for working memory (i.e., Digit Span), planning (i.e., Tower), delay aversion (i.e., Delay of Gratification Task), and EF related behaviors (i.e., BRIEF General Executive Composite) as perceived by parents and teachers, a closer analysis of the structure matrix (cf. Table 3) revealed only three significant predictors, specifically, the BRIEF General Executive Composite—Teacher Form (0.681), the Delay of Gratification Task (−0.587), and the BRIEF General Executive Composite—Parent Form (0.511). Planning (i.e., Tower: number of problems correctly solved at first trial), short-term and working memory (i.e., Digit Span), cognitive flexibility (i.e., Trails: Part B), and attention (i.e., Trails: Part A) were poor predictors of group membership.

The classification results, shown in Table 4, reveal that the discriminant function presents good levels of specificity (100%) and sensitivity (94.1%), with 97.1% of the original grouped cases correctly classified. Only one child in the ADHD group was classified into the TD group. The cross-validated classification showed that, in general, 94.1% of cases were correctly assigned, with one child in each group being misclassified.

## 4. Discussion

The purpose of this study was two-fold: (1) to examine ‘cool’ and ‘hot’ aspects of executive functioning that can lead to a better diagnostic prediction and (2) to evaluate whether participants were adequately assigned to the Attention Deficit/Hyperactivity Disorder (ADHD) group based on the predictors identified. For that purpose, seventeen children with ADHD were matched to 17 typically developing (TD) peers by age, gender, and non-verbal intelligence. Performance-based measures (viz., digit span, tower, trails, and delay of gratification task) and behavioral ratings (viz., BRIEF) were used to assess short-term and working memory, planning, attention, cognitive flexibility, delay aversion, and executive function (EF) related behaviors in everyday life, respectively. Three questions were formulated to fulfill the overall purpose of this study; these will be discussed in the following paragraphs.

### 4.1. In What Dimensions Do Children with ADHD Differ from Children with Typical Development?

Significant differences between the ADHD and TD groups were found in the linear combination of EF measures. Specifically, the groups differed in measures of short-term and working memory, planning, delay aversion, and EF-associated behaviors according to parents and teachers.

The present study confirmed the findings of previous research on working memory impairments in ADHD. In fact, over the years, several studies have shown that working memory performance is impaired in children with ADHD, although different measures have been used to assess this ability. For instance, in their metanalytic review, Martinussen et al. [71] found that children with ADHD showed moderately impaired verbal working memory compared to TD peers. Similar results have been found in the studies of Holmes et al. [14] and Skogli et al. [15], which illustrate a growing consensus suggesting that ADHD represents a group of subjects with working memory impairments.

Our results are also in line with previous research as differences in planning abilities in children with ADHD have been frequently reported (e.g., [17,18,39]). For example, Willcutt et al. [18] pinpointed, in their meta-analysis, that 59% of the included studies reported difficulties in measures of planning. However, several studies conveyed contradicting findings (e.g., [19,20,21,22,23]). For example, studies by Skogli et al. [15], Bünger et al. [24], and Boyer et al. [25] found no difficulties in their samples of children and adolescents with ADHD when assessing planning abilities. These inconsistent results may entail artifacts of the discrepancy of tasks employed and of the variability in the scores used within each task to characterize planning competencies. For example, Boyer et al. [25], Holmes et al. [14], and Skogli et al. [15] applied the same task—the Tower Test of the Delis-Kaplan Executive Function System—and although all used the ‘total achievement score’ as a measure of planning ability, rule violations [14] and the move accuracy ratio [25] were also considered.

Furthermore, our findings replicate and extend previous studies suggesting that children with ADHD are characterized as having a delay-averse motivational style (e.g., [58,59,60]) and that delay aversion may be useful in predicting and diagnosing the disorder. In fact, the preference for immediate outcomes in children with ADHD is one of the most consistent findings in motivational research [51,61]. For example, Solanto et al. [58] applied the Choice Delay Task to children with ADHD aged 7 to 9 years. In this task, the children are required to play a game to earn points that could be exchanged for a nickel. This study demonstrated that children with ADHD exhibit a clear preference for immediate rewards of lower value to the detriment of larger delayed rewards. Coghill et al. [59] found similar results in a sample of clinic-referred medication-naïve (i.e., unmedicated) 6- to 12-year-olds resorting to this same task, with a large effect size (0.82). However, others have found no evidence that delay aversion is a domain of differentiation between children with and without an ADHD diagnosis (e.g., [15,72,73]). For example, Skogli and colleagues [15] evaluated delay of gratification by resorting to the Hungry Donkey Task [74]. In this task, participants help a donkey collect as many apples as possible by selecting four possible doors (A, B, C, and D). When comparing their sample of ADHD (–combined and –inattentive presentation) with a typically developing control group, the authors found that all groups made slightly more advantageous choices (doors C and D) than disadvantageous ones (doors A and B). Furthermore, the authors found interesting results when comparing age groups, since older age was associated with better performance on this task. These contradictory results may be justifiable by age differences. Previous research with typically developing children (aged 6–12 years) has highlighted differences between younger and older age groups, in different variations of the Iowa Gambling Task, with younger children selecting disadvantageous choices more often than older subjects [75]. Additionally, it may be hypothesized that tasks that do not reinforce or penalize the child directly (i.e., gains and penalties within the game do not translate into ‘real’ prizes or losses for the child; for example, the Hungry Donkey Task and some variations of the Iowa Gambling Task as applied to children), do not ‘activate’ motivational processes as strongly as when the child has something to gain or lose directly, possibly giving rise to some of these discrepant results.

Our study did not find significant differences between groups in measures of selective and sustained attention and processing speed resorting to completion time on Trails: Part A. In fact, unimpaired selective attention competencies have been previously reported. Heaton et al. [76] evaluated several subcomponents of attention (i.e., sustained, selective, and attentional control) in 63 children with ADHD, resorting to the Test of Everyday Attention for Children, and found no differences between groups on subtests of selective attention, suggesting that children with this diagnosis have difficulties sustaining and controlling attention. In the study by Mason et al. [77] slower reaction times were found in children with ADHD, but these were not significant relative to nonclinical controls, leading the authors to conclude that children with ADHD have unimpaired mechanisms of visual search. Interestingly, the authors found that the groups differed in the number of errors committed while performing the task. These findings are, partially, in line with those reported in the present study, as differences were not found when assessing selective attention considering completion time on the Trails: Part A. Despite these findings, differences are frequently found in selective attention measures (e.g., [78,79,80]. For example, Kiliç et al. [80] reported that children with ADHD demonstrate impaired performance on the cancellation test, which also evaluates sustained and selective attention. Assef et al. [78] described that children with ADHD exhibit higher reaction times that, in turn, pinpoint selective attention deficits. As stated above, there is a considerable amount of variation in the tasks chosen to assess selective, sustained attention, and processing speed. Not only that, but discrepant scores are utilized between studies (i.e., use of reaction time vs. use of accuracy of response scores), making it difficult to compare results and draw more homogeneous conclusions.

Although difficulties with cognitive flexibility have been previously described (e.g., [14,19,21,26]), we were unable to corroborate these findings considering completion time on the Trails: Part B. Although we are not the first ones to report unimpaired cognitive flexibility competencies in children with ADHD (e.g., [24]), we must consider that our relatively small sample size could have prevented us from finding differences between groups. However, and as previously stated, the different tasks or scores within tasks utilized to characterize this domain could influence the results. For example, Holmes et al. [14] described difficulties with cognitive flexibility on the Trail Making Test, as the ADHD group committed significantly more errors than the control group in the completion of the three conditions. However, the differences in completion time were not significant, which is consistent with the findings derived from the current study. Consequently, it is possible that the number of errors committed during the task is significantly more representative of the child’s cognitive flexibility capacity than the completion times. Furthermore, it has been proposed that the difference between completion times in Trails: Part B and Trails: Part A might be a purer measure of cognitive flexibility [81]. However, in our sample, this particular outcome did not comply with the testing assumptions and, thus, had to be removed from the analysis. Future studies should explore these outcomes and hypothesis.

In the current study, the Global Executive Composite from the Parent and Teacher Forms of the BRIEF was found to differentiate children with and without ADHD. This finding is in line with the conclusions arising from previous research. Several studies to date have reported elevated scores on almost all BRIEF subscales in children with ADHD (e.g., [15,17,42,44]). Although individual subscales were not analyzed in the present study, the current literature reveals that there is a convergence of results for inhibition, working memory, planning/organization (e.g., [17,25,40,42]), and the three main indexes (i.e., Behavioral Regulation Index, Metacognition Index, and General Executive Composite; e.g., [40,42,44]). Both parent and teacher forms of the scale appear to provide relevant information on EF deficits in children with ADHD (e.g., [17,42]).

Overall, there is a high level of heterogeneity in the results of studies evaluating EF in children with ADHD. This incongruity may derive from important methodological constraints such as (a) sample characteristics (e.g., sample size, gender variability, comorbidity patterns), (b) different sampling selection processes (e.g., inclusion and exclusion criteria, diagnostic criteria and formulation), and (c) discrepancy of the assessment materials and analyzed scores (e.g., evaluation of cognitive flexibility resorting to Trail Making Test vs. Wisconsin Card Sorting Task; consideration of completion times vs. accuracy of response). Future studies should try to address these limitations.

### 4.2. How Can the EF Measures Be Combined to Reliably Separate Groups? Based on the Classification Procedure, What Proportion of Children Was Correctly Identified and Classified in Their Group?

In our study, only three of the seven EF measures were considered good predictors of group membership: the BRIEF General Executive Composite—Teacher form, the Delay of Gratification Task, and the BRIEF General Executive Composite—Parent form. The discriminant function analysis (DFA) revealed that these predictors correctly identified and classified 97.1% of the children. Cross-validation revealed a prediction accuracy of 94.1%. These results go beyond previous reports that have evaluated the potential of EF measures to predict group membership in ADHD. For instance, Tan et al. [40] found that none of the performance-based measures employed (viz., WISC-IV: Digit Span Backward, Letter-number Sequencing, Symbol Search, Coding, Working Memory Index, Processing Speed Index, and Continuous Performance Test) predicted group membership above chance levels, even though differences between groups were found on the Conners Continuous Performance Test (CPT) variability score. Additionally, within the BRIEF subscales and indexes, working memory, inhibition, organization of materials, the metacognition index, and the global executive composite accurately predicted group membership by correctly classifying 76.2% of the participants. Results detailed by Toplak et al. [17] also reiterate the importance of behavioral measures as they found that, when performance and behavioral measures were entered together in a logistic regression, only the BRIEF subscales were significant predictors of group membership. Furthermore, according to this study, working memory (96%) and planning (91.9%) were identified as the most accurate predictors [17]. However, it is important to note that acceptable classification levels were obtained by Holmes et al. [14] resorting only to performance-based measures. In fact, the corresponding function derived from the discriminant analysis correctly classified 88.3% of children with ADHD and 81.6% of the control sample.

The discrepancies described above may be related to the sample sizes (e.g., [14]: *n*_ADHD_ = 80; [40]: *n*_ADHD_ = 42), as larger samples are associated with more robust findings and smaller samples are linked to low power to detect differences between groups. In addition, it may be that more comprehensive assessments (i.e., multiple measures to assess a specific function) are necessary to detect differences between groups, and therefore for the accuracy of predictions to become significant. However, our results corroborate the importance of including observational measures of EF in the evaluation of children with ADHD, as the BRIEF—General Executive Composite was found to be a significant predictor of group membership.

Regarding delay aversion, the dual-pathway model postulated by Sonuga-Barke [82] states that EF impairments are more prominent in daily life when affective and motivational processes interact with more ‘pure’ cognitive processes. As such, it is possible that the heterogeneity of the results reported in the literature on performance-based measures arises from their failure to capture important motivational and affective aspects associated with executive functioning in daily life [28]. In the present study, delay aversion was found to be one of the most significant predictors of ADHD, highlighting that children with this clinical disorder tend to prefer smaller rewards obtained immediately over larger rewards obtained after a significant delay in time. Consequently, it may be important to consider this dimension in clinical practice.

### 4.3. Limitations and Directions for Future Studies

There are some limitations to the current study: (1) the small sample size included may lead to low power to detect more robust differences between groups, therefore preventing the identification of important predictors that contribute to diagnostic accuracy; (2) the version of the BRIEF questionnaire used is a translation not yet validated to the Portuguese population; and (3) due to institutional constraints, it was not possible to assess the presence of ADHD symptoms in the control group. Consequently, these constraints limit the generalization of the current findings and, thus, should be carefully considered before being referred for diagnostic confirmation. Future studies should include larger sample sizes to: (a) produce more accurate and robust results; (b) enable the inclusion of other important predictors (e.g., inhibitory control) and/or analyze multiple possible scores achieved within a task to understand which can better contribute to our understanding of ADHD; and (c) analyze the contribution of the BRIEF subscales individually. Additionally, it may be of interest to compare specific BRIEF subscales with performance-based measures that analogously assess the same domains and analyze and compare their accuracy in predicting group membership. Due to the small sample size of our study, the number of predictors entered in the analyzes needed to be reduced. Furthermore, forthcoming studies should place a higher emphasis on ‘hot’ EF and their relationship with cognitive processes.

However, the present study comprises some important strengths: (a) inclusion of a homogeneous sample of children with ADHD (i.e., without comorbid diagnoses); (b) children were matched by age, gender, and non-verbal intelligence; (c) EF questionnaires related to both parent and teacher points of view were analyzed; and (d) inclusion of a measure of ‘hot’ EF, which is frequently lacking in studies assessing EF in children with this diagnosis.

### 4.4. Implications for Clinical Practice

Although several questions remain related to the utility of performance-based measures in the diagnosis of ADHD, its potential to identify difficulties within executive functioning to inform intervention practices is undeniable. Their value in intervention planning is two-fold.

(1) As suggested by Toplak et al. [38], considering the structured environment in which assessments are usually carried out, performance-based measures could provide information regarding the optimal performance of the child within an organized and controlled environment. This can possibly lead to the establishment of individual recommendations on strategies and accommodations that can be employed daily, both at school and at home, to provide more structure and therefore facilitate the completion of tasks and reinforce or extinguish specific behaviors.
(2) If, even in highly structured settings, the child reveals difficulties in executive functioning, the neuropsychological evaluation resorting to performance-based measures will yield information regarding their EF profile and can guide the formulation of an intervention plan that is adapted to the child’s own characteristics and targets specific domains of impairment.

In the current literature, several authors (e.g., [28,45]) posit that the ability of behavioral measures to detect EF difficulties supersedes performance-based measures as they present high levels of ecological validity (i.e., ability of a measure to reflect or predict behavior in everyday life; [83]), producing a more valid representation of the child’s EF competencies in daily life. According to our findings, and findings of previous studies (e.g., [17,40]), these measures are highly predictive of a diagnosis of ADHD and can, therefore, be considered a staple in neuropsychological assessment. However, these questionnaires are not exempt from limitations, as they depend on the subjective opinions of parents and teachers [83]. As, often, children with ADHD present difficulties monitoring and controlling their behavior, high levels of parental and teacher stress (e.g., [84,85]) could hinder their objectiveness. Additionally, as demands and expectations differ between settings and informants, points of view of parents and teachers are usually discrepant and, as such, results should always be carefully inspected.

In summary, evidence retrieved from the use of performance and behavioral measures showcase distinctive levels of executive functioning. Consequently, the clinician should not expect convergence of results between these measures and ought to see them, instead, as cumulative evidence on the presence or absence of EF deficits.

Lastly, considering the potential for delay aversion to differentiate children with and without ADHD symptomatology, it is important to emphasize the importance of measures of ‘*hot*’ executive functioning in the intervention planning process. These could be laboratory measures, such as the Delay of Gratification Task, or information on the child’s motivational and affective competencies collected in a semi-structured interview with parents or caregivers.

Furthermore, ‘hot’ EF have important implications for children’s cognitive processes. For instance, children are presumably more attentive in class if they are motivated to learn and/or enjoy the subject being taught. Contrarily, if the child feels anxious about her school performance and/or perceives the lecture as too difficult considering her perception of her cognitive abilities, her performance will likely be impacted. Thus, considering and understanding the child’s level of reward sensitivity in intervention planning might constitute an important strategy for behavioral modification and symptomatology improvement.

## 5. Conclusions

This study provided important contributions to understanding the neuropsychological, behavioral, and symptomatologic profile of children with ADHD. Children with this diagnosis had impairments in several EF domains, such as short-term and working memory, planning, and delay aversion. These difficulties were evident across settings, as both parents and teachers rated children with ADHD as having higher levels of difficulties in behavior and task-related EF in everyday life, clearly distinguishing them from children without ADHD symptoms. These findings highlight the importance and contribution of both behavioral and ‘hot’ measures of EF for the characterization of ADHD in children. As such, these results may help to establish a basis for the development of non-pharmacological intervention programs, as these several key areas of functioning may be the best targets of intervention.

## Figures and Tables

**Table 1:** Descriptive statistics for age, performance-based, and behavioral measures for participants in the Attention Deficit/Hyperactivity Disorder (ADHD) and typically developing (TD) groups.

	ADHD		TD	
	M	SD	M	SD
Age	8.58	1.22	8.77	1.14
**Non-verbal intelligence**				
Ravens Progressive Colored Matrices	24.53	4.19	25.06	3.91
**Short-term and working memory**				
Digit Span	8.47	2.15	10.94	2.05
**Sustained, selective** **attention** **, and processing speed**				
Trails A (time in seconds)	47.76	17.02	52.94	17.10
**Cognitive flexibility**				
Trails B (time in seconds)	153.76	38.82	126.76	40.82
**Planning and problem solving**				
Tower (correct at 1st trial)	5.94	1.60	7.65	1.06
**Delay aversion**				
Delay Gratification Task	1.82	2.07	6.88	1.97
**Daily executive functioning—BRIEF**				
General executive composite (Parent)	160.65	19.61	121.18	16.44
General executive composite (teacher)	164.00	15.69	107.59	22.52

**Table 2:** Domains assessed by the performance and behavioral measures employed.

Domain	Measure
Non-verbal intelligence	Ravens Colored Progressive Matrices
Auditory short-term and working memory	Digit Span
Selective, sustained attention and processing speed	Trails: Part A (BANC)
Cognitive flexibility	Trails: Part B (BANC)
Planning and problem solving	Tower (BANC)
Delay aversion	Delay Gratification Task
Daily executive functioning	BRIEF

**Table 3:** Structure matrix.

Structure Matrix	
	Function
	1
BRIEF: General Executive Composite—Teacher Form	0.681
Delay Gratification Task	−0.587
BRIEF: General Executive Composite—Parent Form	0.511
Tower: Number of problems correctly solved at 1st trial	−0.295
Digit span	−0.276
Trails: Part B	0.159
Trails: Part A	−0.071

**Table 4:** Classification results.

			Predicted Group Membership	
		Group	TD	ADHD
Original	Count	TD	17	0
		ADHD	1	16
	%	TD	100	0
		ADHD	5.9	94.1
Cross-validation	Count	TD	16	1
		ADHD	1	16
	%	TD	94.1	5.9
		ADHD	5.9	94.1

Percentage of originally cases correctly classified: 97.1%; Cross-validated grouped cases correctly classified: 94.1%|ADHD: Attention-Deficit/Hyperactivity Disorder; TD: Typical development.

## Data Availability

Not applicable.
